# A multicenter, multidisciplinary evaluation of 1701 healthcare professional students’ LGBT cultural competency: Comparisons between dental, medical, occupational therapy, pharmacy, physical therapy, physician assistant, and social work students

**DOI:** 10.1371/journal.pone.0237670

**Published:** 2020-08-13

**Authors:** Dustin Z. Nowaskie, Anuj U. Patel, Ryan C. Fang

**Affiliations:** 1 Department of Psychiatry, Indiana University School of Medicine, Indianapolis, Indiana, United States of America; 2 University of Michigan Medical School, Ann Arbor, Michigan, United States of America; 3 University of Washington School of Medicine, Seattle, Washington, United States of America; University of Birmingham, UNITED KINGDOM

## Abstract

**Background:**

Efforts to characterize healthcare professional students’ lesbian, gay, bisexual, and transgender (LGBT) cultural competency are necessary to recommend educational initiatives. Very few studies have evaluated LGBT cultural competency across multiple healthcare disciplines, and no known studies have included students of other healthcare disciplines such as occupational therapy, pharmacy, physical therapy, and physician assistant.

**Methods:**

Healthcare professional students (N = 1701) at three universities across the United States completed a survey consisting of demographics, experiential variables (i.e., LGBT patients and LGBT curricular hours), and the 7-point Likert LGBT-Development of Clinical Skills Scale (LGBT-DOCSS). LGBT-DOCSS scores, annual LGBT patients, and annual LGBT curricular hours were compared across healthcare disciplines.

**Results:**

While students reported very high Attitudinal Awareness (M = 6.48, SD = 0.92), they endorsed moderate Basic Knowledge (M = 5.54, SD = 1.16) and low Clinical Preparedness (M = 3.78, SD = 1.28). After controlling for several demographic and experiential variables, there were significant differences among healthcare disciplines on LGBT-DOCSS scores, with social work students reporting the highest on all scores, and dental students reporting the lowest on all scores except Clinical Preparedness. There were also significant differences among healthcare disciplines on annual LGBT patients [mean range: 0.57 (dental) to 7.59 (physician assistant)] and annual LGBT curricular hours [mean range: 0.51 (occupational therapy) to 5.64 (social work)]. Experiential variables were significant predictors for Overall LGBT-DOCSS, Clinical Preparedness, and Basic Knowledge (all p < 0.001); LGBT patients was also a significant predictor for Attitudinal Awareness (p < 0.05).

**Conclusions:**

Taken together, significant differences in LGBT cultural competency exist across healthcare disciplines, which may result from inadequate experiences with LGBT patients and LGBT curricular education. Future efforts should consider increasing LGBT patient contact hours and LGBT formal education hours to enhance healthcare students’ LGBT cultural competency.

## Introduction

Lesbian, gay, bisexual, and transgender (LGBT) patients face significant amounts of marginalization in healthcare settings. As many as one in five LGBT-identified patients suffer from discrimination in healthcare environments; [1] a number of others experience high rates of denial of essential medications and may even be exposed to verbal and physical violence during physical examinations, which can subsequently lead to avoidance of care. [2] In general, LGBT people endure higher rates of poor physical health, activity limitations, chronic disease, obesity, mental health conditions (i.e., mood disorders, anxiety disorders, and substance use disorders), and suicidality compared to cisgender, heterosexual people. [3–5] As such, identifying avenues that can decrease negative healthcare encounters may lead to better health outcomes for a substantial number of LGBT patients.

Given that LGBT people face disproportionate rates of poor health conditions and suicide, healthcare professional students are in crucial roles as learners to understand the unique associations between demographics and health risks among the LGBT population and to translate this knowledge into culturally-competent care when they become providers. Additionally, as healthcare continues to become increasingly interprofessional in nature, efforts to characterize students’ attitudes, knowledge, and preparedness towards LGBT healthcare across all healthcare disciplines are necessary in order to recommend both local and national educational initiatives that are specific to each discipline.

Over the past decade, there have been endeavors to integrate LGBT-specific healthcare topics into healthcare professional student curricula by providing intergroup exposure to and education about the LGBT patient population. These methods have been effective in increasing knowledge of LGBT healthcare issues and comfortability as well as decreasing bias towards LGBT people. [6–8] However, there remain significant implicit and explicit biases and varying levels of preparedness among healthcare professional students. [9,10] This gap in LGBT cultural competency is likely a result of the novel and nonobligatory nature of current LGBT educational initiatives as well as the variability of these methods with regard to patient exposure, curricular hours, and curricular content.

Before LGBT healthcare curricula can become standardized, universal, and mandatory, a comprehensive evaluation of current shortcomings in cultural competency, patient exposure, and education among all healthcare professional students across all levels of training is warranted. While some studies have characterized medical [9,11] and social work (SW) [12,13] students’ LGBT cultural competency, these previous studies were limited to sole disciplines and specific levels of training. Only one known study has compared LGBT cultural competency among multiple healthcare disciplines. Greene et al. [10] found that compared to medical and nursing students, dental students felt less comfortable discussing sexual health, were less likely to agree that LGBT content was integrated into their curriculum and that their instructors demonstrated competency, and were less likely to report interest in more LGBT training. These differences were suspected to have resulted from gaps in LGBT health content; however, this conclusion is speculative as LGBT patient exposure and education were not quantified. Additionally, to the best of the authors’ knowledge, no known studies have evaluated LGBT cultural competency of other healthcare disciplines such as occupational therapy (OT), pharmacy, physical therapy (PT), and physician assistant (PA).

In this context, we developed the first known multicenter, multidisciplinary evaluation of healthcare professional students’ levels of LGBT cultural competency. The purpose of this study was to: 1) understand the current gaps in cultural competency and experiential variables (i.e., LGBT patients and LGBT curricular hours) among healthcare professional students in general; 2) determine whether appreciable differences exist in LGBT cultural competency, LGBT patient exposure, and LGBT education among dental, medical, SW, OT, pharmacy, PT, and PA students; and 3) examine how experiential variables influence healthcare professional students’ cultural competency.

## Methods

### Study design, setting, and participants

Since participants could not be identified directly, this study was granted exemption by the Indiana University Institutional Review Board (IRB, Protocol #1903093806), University of Michigan IRB (Protocol #HUM00166371), and University of Washington IRB (Protocol #STUDY00007926). Participation was voluntary and anonymous, and initiation and completion of the survey constituted consent of participation. Utilizing convenience sampling, the survey was emailed to contacts at healthcare professional schools (i.e., the healthcare disciplines dentistry, medicine, OT, pharmacy, PT, PA, and SW) of three different universities across the United States (U.S.), requesting that these contacts forward this email to their current healthcare professional students. One additional follow-up reminder to contacts was emailed, and responses were collected between July and December 2019.

### Variables

A 28-item self-reporting, anonymous, cross-sectional survey of demographics, experiential variables, and the LGBT-Development of Clinical Skills Scale (LGBT-DOCSS) [14] was utilized. Seven demographics consisting of age, gender identity, sexual orientation, race, ethnicity, type of school (“healthcare discipline”), and level of training were collected. Three experiential variables were assessed: 1) how many LGBT patients the students had worked with or cared for (“LGBT patients”); 2) how many hours of LGBT education they had received at their current school (“LGBT curricular hours”); and 3) how many total hours of LGBT education they had received ever (“LGBT total hours”).

The LGBT-DOCSS is an 18-item three-factor structure, interdisciplinary clinical self-assessment for healthcare providers. According to Bidell, [14] the LGBT-DOCSS had good internal consistencies (all *a* > 0.80), test-retest reliability (0.87), and content and discriminant validity. All LGBT-DOCSS items consist of 7-point Likert scales (1 = strongly disagree, 4 = somewhat agree/disagree, 7 = strongly agree). Of the 18 items, eight are reverse scored. An overall mean score averages all items (“Overall LGBT-DOCSS”), while each subscale is composed of the averages of select items (seven “Clinical Preparedness”, seven “Attitudinal Awareness”, and four “Basic Knowledge” items). Higher scores are indicative of higher levels of clinical preparedness and knowledge and less prejudicial attitudes regarding LGBT patients. While the LGBT-DOCSS has not been applied to healthcare professional students broadly, its interdisciplinary utility is promising.

### Statistical methods

Results were analyzed using SPSS Statistics 26 (IBM Corp., Armonk, NY). The number of LGBT extracurricular hours (“LGBT extracurricular hours”) was determined by subtracting LGBT curricular hours from LGBT total hours. Given that training duration of healthcare disciplines varies, the number of annual LGBT patients, annual LGBT curricular hours, and annual LGBT extracurricular hours were determined by dividing LGBT patients, LGBT curricular hours, and LGBT extracurricular hours, respectively, by level of training. Internal consistencies were calculated for LGBT-DOCSS scales, given the novelty of this self-assessment. Demographic frequencies, means, chi-square tests, and one-way analyses of variance (ANOVAs), LGBT-DOCSS score means and analyses of covariance (ANCOVAs), and experiential variable means and ANCOVAs across healthcare disciplines were computed. Paired sample t-tests were conducted to assess differences in LGBT-DOCSS scores. Multiple linear regression models were analyzed to predict LGBT-DOCSS scores based on demographic and experiential variables. Statistical significance was set at *a* = 0.05. Excluded responses were those of missing data.

## Results

### Demographics

A total of 1701 healthcare professional students (dental: 8.4%, medical: 55.3%, OT: 3.7%, pharmacy: 8.1%, PT: 2.5%, PA: 2.5%, and SW: 19.6%) completed the survey (Table 1). The overall response rate was 22.8%; response rates varied across healthcare disciplines (dental: 16.9%, medical: 27.6%, OT: 68.5%, pharmacy: 21.1%, PT: 19.3%, PA: 18.1%, and SW: 16.6%) and universities (university #1: 19.2%, university #2: 29.0%, and university #3: 22.3%). The majority were in their twenties, cisgender women, heterosexual, White/Caucasian, and not Hispanic or Latino. Students were enrolled at three different universities across the U.S. All levels of training were represented, with the exception of pharmacy students from year six. Of note, at these universities, the training duration of healthcare disciplines varied (dental: four years, medical: four to eight years, OT and PA: two years, pharmacy: four to six years, PT: three years, and SW: four to six years). Some medical students (2.7%) were enrolled in dual degree programs, and some SW students (5.1%) were graduate level students. There were significant differences between healthcare disciplines for age [F(6, 1691) = 15.349, p < 0.001], gender [*x*^2^ (30) = 163.357, p < 0.001], sexual orientation [*x*^2^ (30) = 120.954, p < 0.001], race [*x*^2^ (18) = 103.779, p < 0.001], university [*x*^2^ (12) = 471.213, p < 0.001], and level of training [*x*^2^ (36) = 466.369, p < 0.001]. There were no significant differences between healthcare disciplines for ethnicity.

**Table 1:** Demographics.

	M (SD) or n (%)							
	Overall (N = 1701)^a^	Dental (n = 143)	Medical (n = 940)	Occupational therapy (n = 63)	Pharmacy (n = 137)	Physical therapy (n = 42)	Physician assistant (n = 43)	Social work (n = 333)
Age	25.74 (4.39)	25.06 (2.88)	25.49 (2.90)	25.17 (5.80)	23.97 (2.69)	26.24 (4.07)	25.53 (4.54)	27.56 (7.22)
Gender identity								
Cisgender man	480 (28.2%)	59 (41.3%)	344 (36.6%)	3 (4.8%)	34 (24.8%)	8 (19.0%)	7 (16.3%)	25 (7.5%)
Cisgender woman	1191 (70.0%)	82 (57.3%	586 (62.3%)	60 (95.2%)	98 (71.5%)	33 (78.6%)	35 (81.4%)	297 (89.2%)
Non-binary	14 (0.8%)	0 (0.0%)	4 (0.4%)	0 (0.0%)	2 (5.4%)	1 (2.4%)	0 (0.0%)	7 (2.1%)
Transgender man	6 (0.4%)	0 (0.0%)	2 (0.2%)	0 (0.0%)	2 (1.5%)	0 (0.0%)	0 (0.0%)	2 (0.6%)
Transgender woman	2 (0.1%)	0 (0.0%)	2 (0.2%)	0 (0.0%)	0 (0.0%)	0 (0.0%)	0 (0.0%)	0 (0.0%)
Other^b^	8 (0.5%)	2 (1.4%)	2 (0.2%)	0 (0.0%)	1 (0.7%)	0 (0.0%)	1 (2.3%)	2 (0.6%)
Sexual Orientation								
Bisexual	139 (8.2%)	7 (4.9%)	80 (8.5%)	1 (1.6%)	6 (4.4%)	2 (4.8%)	2 (4.7%)	41 (12.3%)
Gay	62 (3.6%)	3 (2.1%)	48 (5.1%)	0 (0.0%)	6 (4.4%)	0 (0.0%)	0 (0.0%)	5 (1.5%)
Heterosexual	1386 (81.5%)	128 (89.5%)	769 (81.8%)	61 (96.8%)	118 (86.1%)	37 (88.1%)	40 (93.0%)	233 (70.0%)
Lesbian	25 (1.5%)	1 (0.7%)	14 (1.5%)	0 (0.0%)	2 (1.5%)	1 (2.4%)	1 (2.3%)	6 (1.8%)
Queer	42 (2.5%)	0 (0.0%)	15 (1.6%)	0 (0.0%)	0 (0.0%)	2 (4.8%)	0 (0.0%)	25 (7.5%)
Other^b^	47 (2.8%)	4 (2.8%)	14 (1.5%)	1 (1.6%)	5 (3.6%)	0 (0.0%)	0 (0.0%)	23 (6.9%)
Race								
Asian/Asian American	253 (14.9%)	35 (24.5%)	158 (16.8%)	5 (7.9%)	35 (25.5%)	0 (0.0%)	2 (4.7%)	18 (5.4%)
Black/African American	73 (4.3%)	5 (3.5%)	30 (3.2%)	2 (3.2%)	3 (2.2%)	1 (2.4%)	0 (0.0%)	32 (9.6%)
White/Caucasian	1234 (72.5%)	87 (60.8%)	674 (71.7%)	55 (87.3%)	85 (62.0%)	40 (95.2%)	40 (93.0%)	253 (76.0%)
Other	141 (8.3%)	16 (11.2%)	78 (8.3%)	1 (1.6%)	14 (10.2%)	1 (2.4%)	1 (2.3%)	30 (9.0%)
Ethnicity								
Hispanic or Latino	111 (6.5%)	6 (4.2%)	67 (7.1%)	2 (3.2%)	7 (5.1%)	1 (2.4%)	2 (4.7%)	26 (7.8%)
Not Hispanic or Latino	1590 (93.5%)	137 (95.8%)	873 (92.9%)	61 (96.8%)	130 (94.9%)	41 (97.6%)	41 (95.3%)	307 (92.2%)
University								
University #1	689 (40.5%)	57 (39.9%)	392 (41.7%)	43 (68.3%)	32 (23.4%)	22 (52.4%)	43 (100.0%)	100 (30.0%)
University #2	661 (38.9%)	66 (46.2%)	257 (27.3%)	0 (0.0%)	105 (76.6%)	0 (0.0%)	0 (0.0%)	233 (70.0%)
University #3	351 (20.6%)	20 (14.0%)	291 (31.0%)	20 (31.7%)	0 (0.0%)	20 (47.6%)	0 (0.0%)	0 (0.0%)
Level of training								
First year	516 (30.3%)	27 (18.9%)	244 (26.0%)	34 (54.0%)	30 (21.9%)	1 (2.4%)	22 (51.2%)	158 (47.4%)
Second year	540 (31.7%)	84 (58.7%)	250 (26.6%)	29 (46.0%)	23 (16.8%)	22 (52.4%)	19 (44.2%)	113 (33.9%)
Third year	324 (19.0%)	22 (15.4%)	226 (24.0%)	0 (0.0%)	37 (27.0%)	19 (45.2%)	0 (0.0%)	20 (6.0%)
Fourth year	241 (14.2%)	10 (7.0%)	195 (20.7%)	0 (0.0%)	34 (24.8%)	0 (0.0%)	0 (0.0%)	2 (0.6%)
Fifth year and above	54 (3.2%)	0 (0.0%)	22 (2.3%)	0 (0.0%)	13 (9.5%)	0 (0.0%)	2 (4.7%)	17 (5.1%)
Other	26 (1.5%)	0 (0.0%)	3 (0.3%)	0 (0.0%)	0 (0.0%)	0 (0.0%)	0 (0.0%)	23 (6.9%)

^a^N = 1701 for all variables except: age (n = 1698).

^b^For “other” categories:

• gender identity: genderfluid (n = 1), gender questioning (n = 1), and other (n = 6).

• sexual orientation: asexual (n = 9), asexual and demisexual (n = 1), asexual & heterosexual (n = 1), asexual & queer (n = 1), bisexual & heterosexual (n = 4), bisexual & lesbian (n = 1), bisexual & lesbian, & queer (n = 1), bisexual & other (n = 1), bisexual & queer (n = 4), demisexual & heteroromantic (n = 1), gay & heterosexual (n = 1), gay & queer (n = 1), heterosexual & queer (n = 2), heterosexual & questioning (n = 2), lesbian & queer (n = 1), multisexual (n = 1), other (n = 10), pansexual (n = 2), pansexual & bisexual (n = 1), pansexual, bisexual, & queer (n = 1), and questioning (n = 1).

### LGBT-DOCSS scores and comparisons

**Fig 1:**
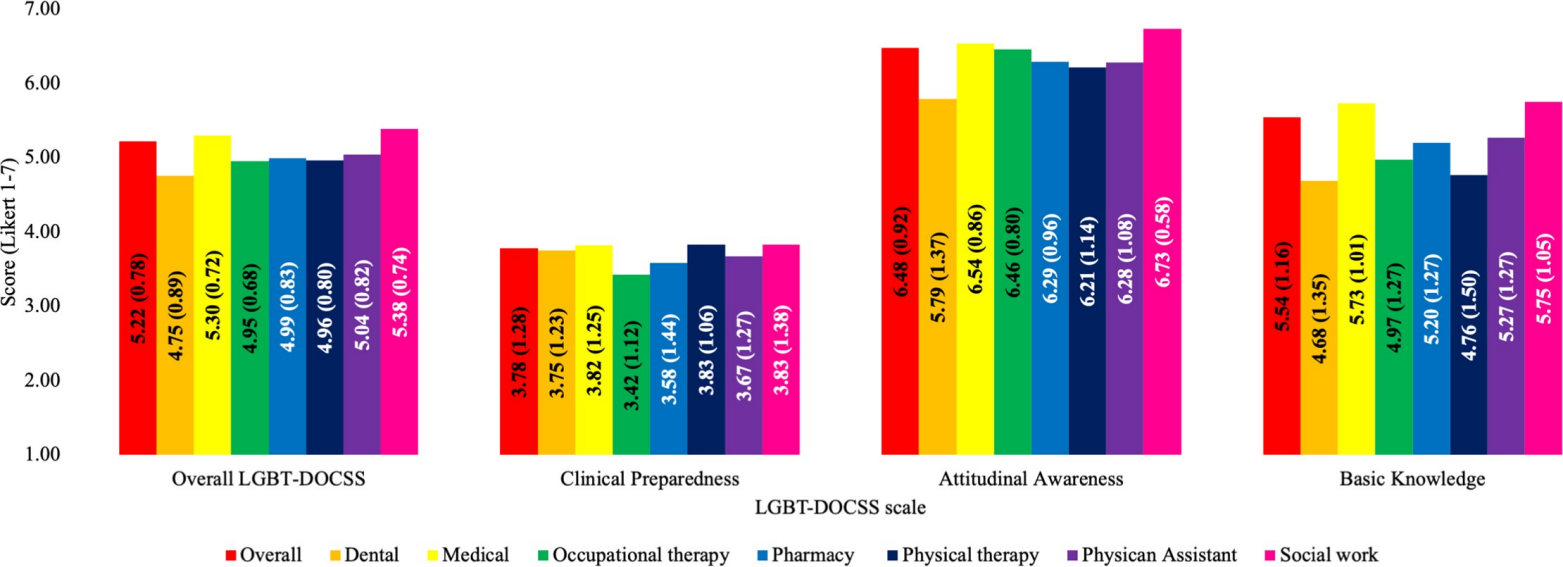
LGBT-DOCSS score means across healthcare disciplines. Abbreviations: LGBT, lesbian, gay, bisexual, and transgender; DOCSS, Development of Clinical Skills Scale. LGBT-DOCSS scores are means on 7-point Likert scales. Higher scores are indicative of higher levels of clinical preparedness and knowledge and less prejudicial attitudes regarding LGBT patients. There were significant differences among healthcare disciplines on LGBT-DOCSS scores, while adjusting for age, LGBT patients, LGBT curricular hours, LGBT extracurricular hours, gender identity, sexual orientation, race, ethnicity, level of training, and university: Overall LGBT-DOCSS [F(6, 1494) = 11.608, p < 0.001], Clinical Preparedness [F(6, 1494) = 4.175, p < 0.001], Attitudinal Awareness [F(6, 1494) = 12.143, p < 0.001], and Basic Knowledge [F(6, 1494) = 21.041, p < 0.001].

Internal consistencies were high for Overall LGBT-DOCSS (*a* = 0.85) and for each subscale (Clinical Preparedness = 0.86, Attitudinal Awareness = 0.90, and Basic Knowledge = 0.82). Considering all healthcare professional students as a whole, the Overall LGBT-DOCSS mean was moderate (Fig 1). Students reported significantly higher Attitudinal Awareness compared to Basic Knowledge [t(1700) = 32.216, p < 0.001] and Clinical Preparedness [t(1700) = 72.513, p < 0.001]; they also reported significantly higher Basic Knowledge than Clinical Preparedness [t(1700) = 50.173, p < 0.001]. After controlling for several demographic and experiential variables, there were significant differences among healthcare disciplines on LGBT-DOCSS scores, with SW students reporting the highest on all LGBT-DOCSS scores, and dental students reporting the lowest on Overall LGBT-DOCSS, Attitudinal Awareness, and Basic Knowledge.

### Experientials and comparisons

**Fig 2:**
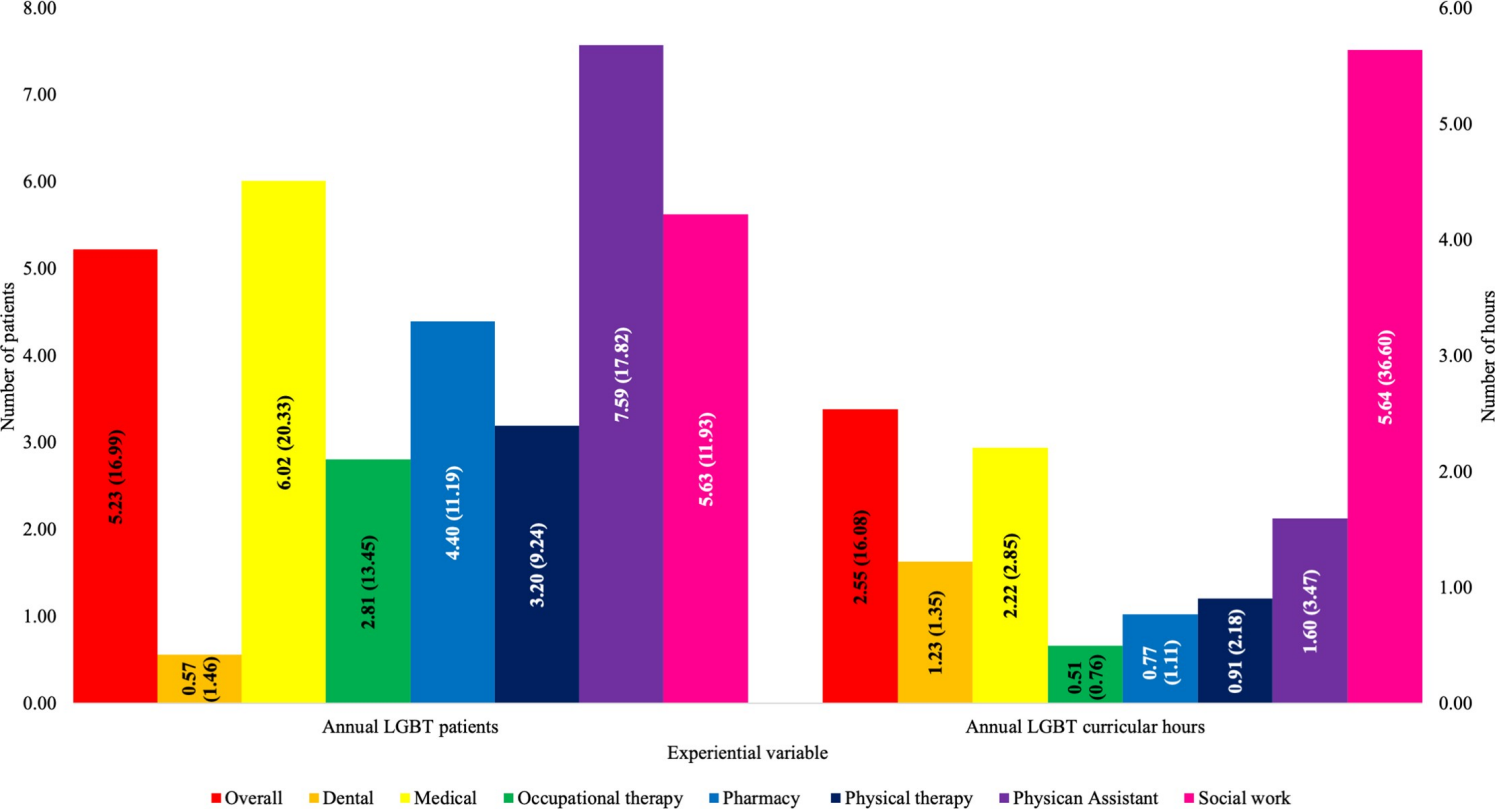
Experiential variable means across healthcare disciplines. Abbreviations: LGBT, lesbian, gay, bisexual, and transgender. There were significant differences among healthcare disciplines on annual LGBT patients, while adjusting for age, LGBT curricular hours, LGBT extracurricular hours, gender identity, sexual orientation, race, ethnicity, healthcare discipline, level of training, and university, F(6, 1495) = 3.155, p = 0.004. There were significant differences among healthcare disciplines on annual LGBT curricular hours, while adjusting for age, LGBT patients, LGBT extracurricular hours, gender identity, sexual orientation, race, ethnicity, healthcare discipline, level of training, and university, F(6, 1495) = 3.105, p = 0.005.

Students reported caring for a wide range of annual LGBT patients and receiving a wide range of annual LGBT curricular hours (Fig 2). After controlling for several demographic and experiential variables, there were significant differences among healthcare disciplines on annual LGBT patients, with PA students having the most patient exposure (7.59 annual patients) and dental students the least (0.57 annual patients), as well as significant differences among healthcare disciplines on annual LGBT curricular hours, with SW students receiving the most education (5.64 annual curricular hours) and OT receiving the least (0.51 annual curricular hours). Additionally, students had also received a moderate number of annual LGBT extracurricular hours: overall (M = 8.31, SD = 34.42), dental (M = 3.28, SD = 7.16), medical (M = 6.93, SD = 24.97), OT (M = 13.71, SD = 38.73), pharmacy (M = 4.18, SD = 21.77), PT (M = 5.15, SD = 16.26), PA (M = 8.31, SD = 21.48), and SW (M = 15.90, SD = 61.62).

### Experiential predictors of LGBT-DOCSS scores

Significant regression equations were found for all LGBT-DOCSS scores with demographic and experiential variables as significant predictors: Overall LGBT-DOCSS [F(11, 1495) = 39.576, p < 0.001, R^2^ = 0.226], Clinical Preparedness [F(11, 1495) = 38.426, p < 0.001, R^2^ = 0.220], Attitudinal Awareness [F(11, 1495) = 18.016, p < 0.001, R^2^ = 0.117], and Basic Knowledge [F(11, 1495) = 14.749, p < 0.001, R^2^ = 0.098]. Concerning experiential variables, LGBT patients and LGBT curricular hours were significant predictors for Overall LGBT-DOCSS, Clinical Preparedness, and Basic Knowledge (all p < 0.001); LGBT patients was also a significant predictor for Attitudinal Awareness (p < 0.05).

## Discussion

To our knowledge, this study presents the first multicenter, multidisciplinary assessment of multiple healthcare professional students’ levels of LGBT cultural competency. Over the past decade, there has been momentum supported by evidence [6,7] to increase LGBT healthcare education. Obedin-Maliver et al.’s [15] and Hillenburg et al.’s [16] comprehensive studies of 176 medical schools and 32 dental schools, respectively, revealed the sparse LGBT education that students across the nation receive. While studies of this nature are becoming increasingly common, much of the current literature regarding LGBT healthcare education has focused on the competency of dental, [10] medical, [9–11] and SW [12,13] students specifically. Given that healthcare continues to become more collaborative and interprofessional, this study aimed to clarify LGBT cultural competency and experiential variables among many healthcare disciplines.

There was a significant difference between reported attitudes and clinical preparedness, with knowledge falling in between. After controlling for many demographic and experiential variables, there were significant differences in LGBT cultural competency, LGBT patient exposure, and LGBT formal education between students of varying healthcare disciplines. Notably, social work students reported the highest on all LGBT-DOCSS scores. These students also reported the highest amount of LGBT curricular and extracurricular education. In contrast, dental students reported the lowest on Overall LGBT-DOCSS, Attitudinal Awareness, and Basic Knowledge. Dental students also received some of the lowest amounts of curricular and extracurricular hours of LGBT education. Furthermore, dental students reported caring for a mere 0.57 LGBT patients per year.

These findings specific to dental students are consistent with Greene et al. [10] and Hillenburg et al. [16] who independently found significant shortcomings in LGBT preparedness and curricular education, respectively. These healthcare discipline-specific discrepancies may be, at least partially, a function of the nature of patient interactions that are specific to each discipline. For example, Greene et al. [10] hypothesized that dental practitioners may operate under the assumption that their practice would not benefit from knowing a patient’s LGBT identity. In reality, the LGBT population faces increased oral health disparities, notably oral human papillomavirus (HPV) prevalence, as well as systemic barriers to receiving dental care. [17] Thus, a dental professional’s awareness of a patient’s LGBT identity could increase vigilance during oral care and may lower a provider’s threshold for HPV testing on suspect lesions.

As noted previously, to the best of the authors’ knowledge, no known studies have assessed LGBT cultural competency of OT, pharmacy, PT, and PA students. Among these healthcare disciplines evaluated in this study, students reported substantially lower Overall LGBT-DOCSS and Basic Knowledge compared to medical and SW students. Furthermore, all of these students interacted with less than 5 LGBT patients annually and received under 1 hour of LGBT curricular education annually (with the exception of PA students). Their experiences were substantially more limited, as compared to medical and SW students who cared for more than 5 annual LGBT patients and received more than 2 hours of annual LGBT curricular education, which may contribute to lower LGBT-DOCSS scores.

Given that experiential variables (i.e., LGBT patients and LGBT curricular hours) were significant predictors for all LGBT-DOCSS scores, it appears that exposure to LGBT patients and LGBT curricular education are important factors for healthcare professional students’ cultural competency. This finding is akin to the few studies that have shown that curricular education can be effective in ameliorating LGBT-specific attitudes, preparedness, and knowledge among dental, [18] medical, [7,8] and pharmacy [19,20] students. As such, both local and national educational initiatives should advocate for these experiential variables. Recommendations to increase LGBT patient exposure include delivering panel discussions, establishing seminars and conferences, integrating students into LGBT healthcare clinics, and institutionally promoting safe spaces for LGBT patients. Ways to increase LGBT curricular hours include instituting journal clubs, increasing standard lectures, hosting culturally-competent guest speakers, and identifying extracurricular free, online modules.

Currently, the quantity and quality of LGBT patient exposure and education necessary to achieve high LGBT cultural competency is unknown. Future studies are required to replicate these study results, elucidate the amount of LGBT patient exposure and education that are required to obtain adequate cultural competency per healthcare discipline, further examine the long-term effects that increased LGBT patient exposure and curricular education have on LGBT cultural competency, and ultimately mandate standardized cultural competency trainings for healthcare professional students.

### Limitations

There are a few notable limitations of this study. First, this study relied on convenience sampling. While recruitment was initially via contacts at each healthcare professional school, it was not determined if all students received the study email. Contacts with biases toward sexual and gender minorities may have received the email and chosen not to distribute the survey to all students because of these biases. Second, students with biases toward sexual and gender minorities may have chosen not to participate as a result of these biases, and LGBT students may have been more likely to respond. Third, these students represent only three universities across the country, and it is unknown whether they are representative of the healthcare professional student population as a whole. Thus, the generalizability of these results is unknown. Fourth, while many dental, medical, pharmacy, and SW students were polled, this study recruited less OT, PT, and PA students and consequently the generalizability of these students is less reliable. Fifth, it is unknown how accurate students were at quantifying the experiential variables used in these analyses. Sixth, while this study assessed the quantity of education hours, it did not evaluate self-reported quality of that education (i.e., quality may be as important as quantity). Finally, while the LGBT-DOCSS’s validity is documented in Bidell’s heterogeneous population of undergraduate (psychology and counseling) and graduate (psychotherapy, counseling, and medical) students from the U.S. and United Kingdom, its applicability to other types of healthcare professional students within dental, pharmacy, occupational therapy, physical therapy, physician assistant, and social work healthcare disciplines has only been documented here. Of note, the psychometric properties and data within this student population are similar to those reported by Bidell. [14]

## Conclusions

Our data represents the first multicenter, multidisciplinary study to assess many types of healthcare professional students’ levels of LGBT cultural competency. Overall, healthcare professional students have high LGBT affirming attitudes, moderate basic knowledge, and low clinical preparedness. Significant differences in cultural competency exist across healthcare disciplines, which may be a result of inadequate experiences with LGBT patients and curricular education. Given existing healthcare disparities of the LGBT population in conjunction with low self-reported clinical preparedness by healthcare professional students, future efforts should consider increasing LGBT patient contact hours and LGBT formal education hours to enhance healthcare students’ cultural competency in the provision of care, treatment, and services for LGBT patients.
